# Air quality and attributable mortality among city dwellers in Kampala, Uganda: results from 4 years of continuous PM_2.5_ concentration monitoring using BAM 1022 reference instrument

**DOI:** 10.1038/s41370-024-00684-9

**Published:** 2024-06-15

**Authors:** Lynn M. Atuyambe, Samuel Etajak, Felix Walyawula, Simon Kasasa, Agnes Nyabigambo, William Bazeyo, Heather Wipfli, Jonathan M. Samet, Kiros T. Berhane

**Affiliations:** 1 Department of Community Health and Behavioural Services, Makerere University School of Public Health, College of Health Sciences, Kampala, Uganda; 2 The Eastern Africa GEOHealth Hub, Kampala, Uganda; 3 Department of Disease Control and Environmental Health, Makerere University School of Public Health, Kampala, Uganda; 4 Department of Epidemiology and Biostatistics, Makerere University School of Public Health, Kampala, Uganda; 5 University of Southern California, Los Angeles, CA USA; 6 Colorado School of Public Health, University of Colorado, Aurora, CO USA; 7 Department of Biostatistics, The Mailman School of Public Health, Columbia University, New York, NY USA

**Keywords:** Air pollution, Health impact, Kampala city, Uganda

## Abstract

**Background:**

Air pollution is a known risk factor for non-communicable diseases that causes substantial premature death globally. Rapid urban growth, burning of biomass and solid waste, unpaved sections of the road network, rising numbers of vehicles, some with highly polluting engines, contribute to the poor air quality in Kampala.

**Objective:**

To provide evidence-based estimates of air pollution attributable mortality in Kampala city, with focus on ambient fine particulate matter (PM_2.5_).

**Methods:**

We utilized a time series design and prospectively collected data on daily ambient PM_2.5_ concentration levels in micrograms per cubic meter (μg/m^3^) using a Beta Attenuation Monitor (BAM-1022) in Kampala city, Uganda. We combined the PM_2.5_ data with all-cause mortality data obtained from the Uganda Bureau of Statistics and the Ministry of Health in Kampala. We calculated attributable risk estimates for mortality using the WHO AirQ+ tools.

**Results:**

Overall, the annual average concentration for PM_2.5_ for the period of 4 years, 2018–2021, was 39 μg/m^3^. There was seasonal variation, with the rainy season months (March–June and October–December) having lower values. PM_2.5_ concentrations tend to be highest in the morning (09.00 h) and in the evening (21.00 h.) likely due to increased vehicular emissions as well as the influence of weather patterns (atmospheric temperature, relative humidity and wind). Saturday has the most pollution (daily average over 4 years of 41.2 μg/m^3^). Regarding attributable risk, we found that of all the deaths in Kampala, 2777 (19.3%), 2136 (17.9%), 1281 (17.9%) and 1063 (19.8%) were attributable to long-term exposure to air pollution (i.e., exposure to PM_2.5_ concentrations above the WHO annual guideline of 5 μg/m^3^) from 2018 to 2021, respectively. For the 4 years and considering the WHO annual guideline as the reference, there were 7257 air pollution-related deaths in Kampala city.

**Impact:**

Our study is the first to estimate air pollution attributable deaths in Kampala city considering the target as the WHO annual guideline value for PM_2.5_ of 5 μg/m^3^. Our monitoring data show that fine particulate matter air pollution in Kampala is above the WHO Air Quality Guideline value, likely resulting in substantial adverse health effects and premature death. While further monitoring is necessary, there is a clear need for control measures to improve air quality in Kampala city.

## Introduction

Ambient air pollution, primarily generated by human activities, is a known causal risk factor for non-communicable diseases and premature death [1]. The resulting disease burden is substantial, making air pollution a leading contributor to the global disease burden and a challenge to advancing public health. In a recent study that used an updated risk relationship for air pollution and mortality [2], particulate matter air pollution from fossil fuel combustion was estimated to be responsible for an estimated 10.2 million premature deaths in 2012, or almost 20% of all global deaths, with about 94% of these occurring in low-and middle-income countries (LMICs). In Africa, where rapid development and urbanization are leading to steadily increasing emissions, air pollution is estimated to have caused 1.1 million premature deaths in 2019 [3]. This figure constitutes more deaths than from tobacco, alcohol, road accidents, and drug abuse combined [3, 4]. Much of Africa faces emissions from household biomass fuel, windblown dust, and agriculture along with rising vehicle traffic and industrialization. Air pollution in Africa, particularly Sub-Saharan Africa, is receiving increasing attention, but monitoring data are limited and epidemiological evidence on health risks in the region remains sparse. Available monitoring data indicate that levels of airborne particulate matter (PM) substantially exceed the World Health Organization’s Air Quality Guidelines [5]. Aggressive programs to improve ambient air quality are lacking.

To provide policy-relevant evidence and to advance air pollution control, the Eastern Africa GEOHealth Hub, hereafter referred to us the Hub, was established in 2015 as a collaborative partnership between academic institutions in Ethiopia, Uganda, Kenya, and Rwanda and in the United States. As a starting point for characterizing air pollution in the four Eastern African countries, Hub investigators installed BAM-1022 ground level monitors for airborne fine particulate matter (PM_2.5_) in four capital cities (Addis Ababa, Ethiopia; Nairobi, Kenya; Kigali, Rwanda; and Kampala, Uganda). With these monitors, the Hub aimed to prospectively monitor PM_2.5_ levels, characterize the daily and seasonal trends of PM_2.5_, and assess the association of exposure to PM_2.5_ concentration with childhood respiratory health, hospitalization, and mortality in these major African cities.

To describe trends of air pollution, to characterize its health impact and to advance its control in Uganda, this paper reports on the PM_2.5_ concentrations measured over 4 years (2018–2021) in Kampala and estimates the associated mortality burden. It complements a similar report for Addis Ababa based on 3 years of monitoring data [6]. The results contribute novel primary data on levels and trends of ambient air pollution in Kampala, Uganda, where health systems are challenged and exposure to air pollution is not routinely monitored. As such, it offers the first evidence-based estimates of air pollution associated mortality in the city. Air quality management in Kampala is challenged by rapid urban growth and the city’s physical characteristics and meteorology. Our findings have the goal of adding to the scientific foundation for air quality control in Uganda.

## Materials and methods

### Design

This study uses data on hourly ambient PM_2.5_ concentration levels in micrograms per cubic meter (μg/m^3^) made using a Beta Attenuation Monitor (BAM-1022). The associated mortality burden is estimated using the World Health Organization’s AirQ+ tool [7].

### PM_2.5_ monitoring

#### Study site

This study was conducted in Kampala, the capital city of Uganda with an estimated population of 1,709,900 [8]. It covers an area of 189.3 square kilometers and has an estimated population density of 9352 per square kilometer. There are two distinct seasons characterized by high and low rainfalls. The dry seasons are June to August and December to February while the wet seasons are March to May and September to November. The PM_2.5_ sampling site was located on the roof of the Makerere University School of Public Health (MakSPH) within Mulago National Referral Hospital complex in Kampala city.

##### Data collection and instrument maintenance

The PM_2.5_ data utilized came from the continuous real-time measurement of PM_2.5_ concentration by a BAM-1022. The BAM-1022 has an internal working environment that maintains a steady Flow Rate (FR) (at 15.8 < FR < 17.5 liters per minute) and a range of meteorological conditions: Relative Humidity (RH) (10% < RH < 99%), Temperature (T) (1 < T < 40 °C), and Barometric Pressure (BP) (650 < BP < 670 mm Hg). After exploring the variability of PM_2.5_ concentrations, data were averaged over 1-h intervals, yielding 24 data points daily. For quality control, the instrument provides error messages so that problems could be addressed when needed and the BAM 1022 was carefully maintained.

**Fig. 1:**
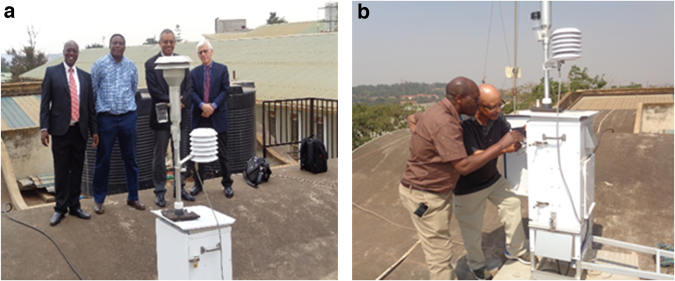
BAM 1022 reference instrument. **a** Investigators' site visit to Makerere University School of Public Health rooftop. **b** Ugandan Principal Investigator with the Air Quality Management District (AQMD) officer performing a quality assurance check.

Our team regularly performs background PM_2.5_ concentration calibration offset in ug/m^3^. The background value is a correction offset for the concentration data collected by the BAM-1022. The background PM_2.5_ concentration calibration offset is factory-calibrated but also during the BAM 1022 set up this value is verified and adjusted if necessary) prior to first use and then every 6 months using the BX-302 Zero Filter Calibration Kit. This test corrects the background PM_2.5_ concentration calibration offset (BKGD) value to compensate for minor variations caused by local conditions such as grounding and shelter characteristics. To measure PM_2.5_, a vacuum pump draws airborne particulate matter of 10 microns and less through a size-selective inlet (the PM_10_ inlet head), down into a PM_2.5_ very sharp cut cyclone where separation of airborne particulate matter of size greater than 2.5 microns takes place, allowing airborne PM of size ≤2.5 micrograms to move down the inlet tube and deposit on a filter tape located between the beta source and detector. The accumulation of mass onto the filter tape increasingly attenuates beta ray transmission through the media. This study used data for the period 1st January 2018 to 31 December 2021 (Fig. 1).

### Mortality data source

We obtained mortality data from the Uganda Bureau of Statistics (UBOS) as well as the District Health Information Software 2 (DHIS2), Division of Health Information Management Ministry of Health, Uganda.

### Data management and quality assurance

Data were downloaded weekly from the BAM-1022 and evaluated according to the Hub’s standard operating procedure. For analysis, we used data for days with at least 18 of 24 h available. The missing data primarily resulted from power outages and periods of routine maintenance. Missing data were detected in the system software records by a signal for a sampling error resulting from data outside of the acceptable ranges for the following selected parameters: flow rate, concentration, temperature, barometric pressure, relative humidity, and sampling time interruption.

Among the techniques proposed in the literature for replacing missing values, we selected substitution of the mean, an imputation technique often used for air pollution data [9]. We adapted the “before-after-mean” method, which replaces all missing values with the mean of one datum before the missing value and one datum after the missing value If at least one of the before-and-after data points were not available, we moved to a 2-day window to complete the data. As a result of this data cleaning process, we recovered 120 days (8.2%) of the expected data.

#### Data analysis

##### Exposure PM_2.5_ data analysis

The averaged continuous ambient PM_2.5_ measurements were used for statistical analysis. The R statistical software (R 3.6.2; https://www.r-project.org/) was used to conduct descriptive analyses. The 1-h BAM 1022 data were aggregated to create the daily averaged data sets for PM_2.5_. Time series line graphs were used to explore daily patterns and seasonality.

##### Attributable death estimation

We used the WHO AirQ+ tool to calculate the deaths attributable to PM_2.5_ [7]. We employed averaged concentrations over the 4 years from the BAM-1022 for the calculation of attributable mortality. The total population of Kampala for the 4 years from 2018 to 2021 was provided by the Uganda Bureau of Statistics (UBOS).

#### Input variables

We obtained the required input data for the AirQ+ tool kit from a variety of sources. These variables included Kampala area (189.3 square kilometers of land and water), population density (9352 per square Kilometer), and Kampala GPS coordinates (0°18′ 49″ N, 32° 34′ 52″ E). The proportion of the population above 30 years was ascertained, and the number of accidental deaths subtracted before arriving at the annual mortality data for use in the AirQ+ tool kit. Deaths resulting from accidents (external causes) are excluded because they are not likely to be related to air pollution. The annual mortality data was captured from the UBOS and the rest of data for all health facilities in Kampala city was obtained from the Ministry of Health of Uganda, Health Information Management Division. Annual PM_2.5_ was computed based on the daily PM_2.5_ captured using the BAM 1022 (Table 1).

**Table 1:** Input variables for health impact assessment of air pollution.

Year	Population			Deaths in the calendar year		Annual PM_2.5_ concentrations	
	Total population	Proportion above 30 years	The number above 30 years	Total	Minus Emergency ward^a^	Before validation and imputation	After validation and imputation
2018	1,620,600	25%	405,150	15,485	14,372	40.7 µg/m^3^	39.3 µg/m^3^
2019	1,650,800	25%	412,700	13,394	11,961	37.7 µg/m^3^	34.9 µg/m^3^
2020	1,680,600	24%	403,344	9491	8070	37.8 µg/m^3^	37.4 µg/m^3^
2021	1,709,900	24%	410,376	7766	5566	41.7 µg/m^3^	42.0 µg/m^3^

^a^Total death excluding death from Emergency ward.

The four WHO annual interim target options for PM_2.5_ (Interim target 1—35 μg/m^3^, interim target 2—25 μg/m^3^, interim target 3—15 μg/m^3^, and interim target 4—10 μg/m^3^) and the WHO annual average air quality guideline for PM_2.5_ (5 μg/m^3^) were used as cut-off reference values to estimate the excess deaths attributable to PM_2.5_ pollution as measured by the BAM 1022. The cut-offs used to estimate the mortality due to air pollution were adopted from the AirQ+ software tool and the interim targets from the WHO global air quality guidelines [1].

## Results

Over the 4 years of the study, data were available for 1069 days, representing 73.2% completeness. The main reasons for missing data were power outages and pump failure. In addition, due to COVID-19 lockdown in Uganda, we missed regular service of the BAM 1022, resulting in pump failure in June and July 2020 (Supplementary Table 1).

The overall annual mean PM_2.5_ concentration for the 4 years (2018–2021) was 39 μg/m^3^, eight times higher than the WHO PM_2.5_ annual average guideline of 5 μg/m^3^. Seasonal variations are apparent with the rainy season months of March–June and October–December recording lower PM_2.5_ concentrations (Supplementary Table 2).

**Fig. 2:**
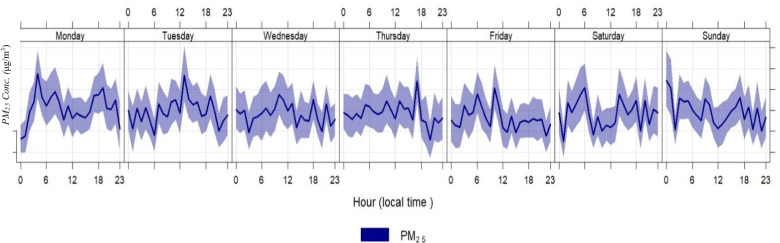
The daily pattern of hourly average PM_2.5_ concentrations in Kampala, Uganda, BAM 1022 (2018–2021).

In this study, we observed that the hourly PM_2.5_ concentrations in Kampala exhibited distinct day-of-week, and seasonal patterns of variation. Fine particulate matter concentrations tend to be highest in the morning (09.00 h) and in the evening (21.00 h) likely due to increased vehicular traffic and emissions at these times. This pattern is also influenced by meteorology (weather patterns, atmospheric temperature, relative humidity, and wind) (Fig. 2).

Over the 4 years (2018–2021), Saturday had a higher PM_2.5_ average concentration with a daily average of 41.2 μg/m^3^. This is the day when most people who use public transport during the week drive into the city for various reasons such as shopping as well as public servants who return to their families in the city. Also, on weekends people from other cities travel to Kampala city (Table 2).

**Table 2:** Day of the week 24-h PM_2.5_ concentrations measured by the BAM 1022.

Year	Weekday							Overall mean
	Sunday	Monday	Tuesday	Wednesday	Thursday	Friday	Saturday	
2018	36.7	37.0	38.5	40.0	42.3	39.9	40.6	39.3
2019	38.3	34.8	33.8	34.3	32.8	32.8	37.8	34.9
2020	36.2	34.8	37.3	38.3	39.1	38.2	37.6	37.4
2021	40.5	42.7	42.3	40.8	39.7	41.5	46.7	42.0
Overall	38.0	37.7	38.4	38.7	39.1	38.7	41.2	38.8

The overall annual mean of PM_2.5_ concentration between 2018–2021 was 38.8 μg/m^3^ with a standard deviation of 18.6 in the range of 1.2–162.9. There was an increase in the average annual PM_2.5_ concentration levels from 2019 to 2021.

### Daily time series: BAM-1022

**Fig. 3:**
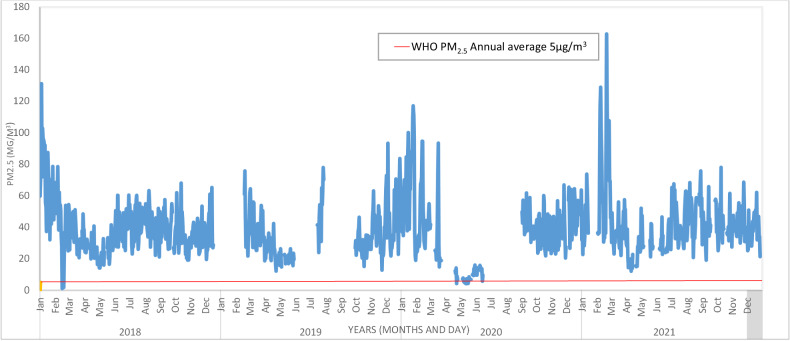
Time series patterns of PM_2.5_ based on BAMs 1022 at Makerere University School of Public Health, 2018–2021.

The daily time series of PM_2.5_ values plotted in Fig. 3 shows that December, January, and February stand out with the highest concentration above 80 μg/m^3^. We also observed that in each year, the lowest PM_2.5_ concentrations were recorded in the months of April and May. The highest recorded PM_2.5_ concentration was in the month of February 2021 during the dry season.

### The health impact of long-term exposure to PM_2.5_ pollution

The attributable burden of premature death due to air pollution as indexed by PM_2.5_ is presented in Table 3. Of the deaths estimated to have occurred in Kampala in 2020 and 2021, 1281 (17.9%) and 1063 (19.8%) non-accidental deaths could be attributed to long-term exposure to air pollution (exposure to PM_2.5_ concentrations above the WHO annual mean of 5 μg/m^3^), respectively. The burden of attributable deaths in Kampala City over the 4 years is estimated to be 7257 when benchmarked against the WHO Air Quality Guideline value of 5 μg/m^3^.

**Table 3:** Estimated annual attributable death from long-term exposure to PM2.5 concentrations (2018–2021).

Year	Annual mean PM_2.5_ concentration, (μg/m^3^)	Annual attributable deaths with 95% CI *N* (%)									
		WHO annual Interim 1 (35 μg/m^3^)		WHO annual Interim 2 (25 μg/m^3^)		WHO annual Interim 3 (15 μg/m^3^)		WHO annual Interim 4 (10 μg/m^3^)		WHO annual Mean (5 μg/m^3^)	
		*N* (95% CI)	% = *N*/^a^	*N* (95% CI)	% = *N*/^a^	*N* (95% CI)	% = *N*/^a^	*N* (95% CI)	% = *N*/^a^	*N* (95% CI)	% = *N*/^a^
2018	39.3 µg/m^3^	480 (320–640)	3%	1300 (860–1700)	9%	2100 (1400–2700)	14%	2400 (1600–3100)	17%	2800 (1900–3600)	19%
2019	34.9 µg/m^3^	190 (130–260)	2%	880 (580–1200)	7%	1500 (1000–2000)	13%	1800 (1200–2400)	15%	2100 (1400–2700)	18%
2020	37.4 µg/m^3^	120 (78–160)	2%	530 (350–690)	7%	530 (350–690)	13%	1100 (740, 1400)	15%	1300 (860–1600)	18%
2021	42.0 µg/m^3^	210 (140–280)	4%	510 (340–670)	10%	790 (530–1000)	15%	930 (630–1200)	18%	1100 (720–1400)	20%

^a^Total death excluding death from Emergency ward in Table 1; Figures rounded off to 2 significant numbers.

## Discussion

We used a high-quality reference monitor (BAM 1022) to continuously measure PM_2.5_ for over 4 years (2018–2021) in Kampala, Uganda with support from the Eastern Africa GEOHealth Hub [10]. We established that ambient levels of PM_2.5_ were two and eight times higher than WHO’s daily and annual mean value guidelines, respectively. Our health impact assessment calculations based on these PM_2.5_ levels revealed that 17–19% of all deaths in Kampala between 2020–2021 were attributable to PM_2.5_ related air pollution. The high mortality burden due to PM_2.5_ underlines the urgent need for Uganda’s policymakers and public health advocates to take immediate measures to control emissions and reduce exposures to the city’s residents.

As observed in several PM_2.5_ monitoring studies in other countries, our study showed seasonal variation in PM_2.5_ with lower air pollution concentrations during the wet seasons (March–May and September–November) [6, 11, 12]. The dry seasons in Kampala are characterized by higher dust levels from unpaved roads, construction, and burning of biomass and plastics [13, 14]. This combustion mix partially explains the corresponding high pollution levels. For the vulnerable populations with respiratory and heart conditions, strategies should be considered for protection, including respiratory protective devices, e.g., N95 respirators, when in dusty and smoky environments in the dry seasons or taking other pragmatic precautions to avoid unnecessary exposure, if possible, e.g., limiting timing in heavily trafficked areas. The attributable burden points to the need for regulation and strict enforcement of the law in Uganda.

We also observed a wide range between the daily maximum and minimum PM_2.5_ concentrations, emphasizing that some hours of the day, notably early morning and evening, have relatively more polluted air. Daily peaks in pollution correspond to the times when most vehicles are on the road, pointing to the substantial contribution made by vehicle exhaust and non-exhaust emissions (mainly brake, tyre wear, and road dust from busy traffic and unpaved roadways) toward ambient air pollution. Air pollution due to motor vehicle emissions is a challenge, especially in low- and middle-income countries where urban populations are growing rapidly, fuel quality is poor, and vehicles are older and irregularly maintained. Our results emphasize the urgent need to establish and strengthen policies aimed at reducing vehicle pollution, including better enforcement of vehicle emission standards, improvements in public transportation and investment in electric vehicles. A system for enforcement and monitoring of regular vehicular servicing and repairs should be developed in collaboration with motor vehicle servicing centers/fuel stations. Such measures would empower relevant governmental bodies such as the Ministry of Works and Transport and the Uganda Police Force to help in enforcing standards given that regular and timely maintenance of car engines reduces emissions and improve air quality.

Daily variation in air pollution could also be influenced by industries operating at night, and at times illegally, when electricity is cheaper and there is less monitoring by regulatory bodies responsible for enforcing the standards set by the Uganda National Environmental Management Authority (NEMA). Household Air Pollution (HAP), mainly from emissions from indoor cooking, could also play a role in increasing fluctuations in pollutant levels as biomass fuel burning for cooking increases emissions in the morning and evening. Other studies have found that increased levels of outdoor pollution in African countries include a contribution from indoor sources [3].

Our burden estimates indicate that the health impact of long-term exposure to PM_2.5_ is likely severe, especially for vulnerable populations such as the elderly, and those with existing non-communicable diseases. Our estimates, while high, only account for outdoor PM_2.5_ pollution levels over the 4 years of continuous monitoring. The combined exposures to air pollution the Ugandan public experiences result from a myriad of indoor and outdoor sources, complicating the challenge of reducing the health effects of air pollution. Other countries in a similar context have also seen rising mortality and morbidity because of indoor and outdoor air pollution [6, 15, 16]. There is an urgent need for stakeholders including the Kampala Capital City Authority (KCCA) Health and Environment Department, KCCA directorates of Directorate of Physical planning, that of Engineering and Technical Services, Uganda National Roads Authority, National Environmental Management Authority, Ministry of Health, Ministry of Water and Environment, bilateral and Multilateral agencies, and the CSO to take action to control levels of air pollution. We anticipate a significant benefit to public health.

This study has several strengths. Using United States Environmental Protection Agency approved PM_2.5_ monitors, the real-time temporal measurements and rigorous quality control have contributed to the collection of 4 years of valid high-quality data. One limitation of our study is data loss from the technical malfunctioning of the BAM 1022 due to lack of regular maintenance during the COVID-19 lockdown in March 2020. At times, we also lost data because of power outages.

## Conclusion

Overall, the findings show that levels of fine particulate matter in Kampala are above the WHO Air Quality Guideline values, resulting in substantial adverse health effects and premature mortality. While further monitoring is necessary, there is a clear need for improved air quality control in Kampala and other similarly growing cities. Poor solid waste management practices such as burning solid waste at household and institutional levels should be stopped, while reducing, reusing, and recycling solid waste, especially plastics, should be promoted. At household and institutional levels, the government should promote interventions such as cooking using clean energy fuels by subsidizing prices of electricity and Liquefied Petroleum Gas (LPG). Electric and gas-based cookers should be promoted by markedly reducing taxes on them. Vulnerable and highly exposed populations, such as those suffering from chronic diseases and those in occupational high-risk groups (e.g., traffic police), should be encouraged to wear respiratory protection during the early morning and evening hours and on dusty roads in the dry seasons. In terms of traffic-related pollution, mass vehicular transport, such as clean fuel buses, should be instituted; all vehicles should be monitored, and high polluting vehicles should not be allowed on the road; and traffic rules should be consistently enforced. Such a multipronged policy approach to reducing air pollution can and would reduce exposures and save lives.

## Supplementary information

Supplementary Table 1

Supplementary Table 2

Supplementary Table 3

reproducibility-and-quality-checklist

## Data Availability

The data generated and analyzed during this study can be made available by the Makerere University School of Public Health Research and Ethics Committee on request at sphrecadmin@musph.ac.ug or the corresponding author.
