# Supplementary material for: Air quality and attributable mortality among city dwellers in Kampala, Uganda: results from 4 years of continuous PM2.5 concentration monitoring using BAM 1022 reference instrument
Source: J Expo Sci Environ Epidemiol. 2024 Jun 15;35(2):288–93. doi: 10.1038/s41370-024-00684-9 (PMC11840866; doi:10.1038/s41370-024-00684-9)
Supplement: Supplementary file 1 — Supplementary Table 1 [file 41370_2024_684_MOESM1_ESM.docx]

Supplemental Table 1. Completeness of the data from the GEOHealth Hub MakSPH BAM 1022

| Years | Month | | | | | | | | | | | | Total |
| --- | --- | --- | --- | --- | --- | --- | --- | --- | --- | --- | --- | --- | --- |
|  | *Jan* | *Feb* | *Mar* | *Apr* | *May* | *Jun* | *Jul* | *Aug* | *Sep* | *Oct* | *Nov* | *Dec* |  |
| 2018 | 31 | 28 | 29 | 27 | 26 | 30 | 28 | 31 | 28 | 31 | 30 | 17 | 336 |
| 2019 | -- | 11 | 31 | 30 | 25 | -- | 14 | -- | 1 | 31 | 30 | 31 | 204 |
| 2020 | 31 | 28 | 15 | 7 | 25 | 15 | -- | -- | 29 | 31 | 27 | 23 | 231 |
| 2021 | 16 | 27 | 31 | 26 | 13 | 21 | 31 | 27 | 22 | 27 | 30 | 27 | 298 |
| Overall | **78** | **94** | **106** | **90** | **89** | **66** | **73** | **58** | **80** | **120** | **117** | **98** | **1,069** |

*Key: -- = Missing data either due to power outage or BAM 1022 Malfunctioning*
