# Supplementary material for: Air quality and attributable mortality among city dwellers in Kampala, Uganda: results from 4 years of continuous PM2.5 concentration monitoring using BAM 1022 reference instrument
Source: J Expo Sci Environ Epidemiol. 2024 Jun 15;35(2):288–93. doi: 10.1038/s41370-024-00684-9 (PMC11840866; doi:10.1038/s41370-024-00684-9)
Supplement: Supplementary file 2 — Supplementary Table 2 [file 41370_2024_684_MOESM2_ESM.docx]

Supplemental Table 2. Monthly PM_2.5_ averages

| Years | Month | | | | | | | | | | |  | Annual mean |
| --- | --- | --- | --- | --- | --- | --- | --- | --- | --- | --- | --- | --- | --- |
|  | *Jan* | *Feb* | *Mar* | *Apr* | *May* | *Jun* | *Jul* | *Aug* | *Sep* | *Oct* | *Nov* | *Dec* |  |
| 2018 | 69.2 | 39.8 | 36.7 | 25.7 | 25.8 | 39.9 | 41.8 | 42.0 | 38.8 | 34.9 | 34.9 | 37.2 | 39.3 |
| 2019 | --- | 41.4 | 38.9 | 25.4 | 20.3 | --- | 50.6 | --- | 26.8 | 29.1 | 36.3 | 47.3 | 34.9 |
| 2020 | 64.2 | 45.5 | 33.0 | 8.5 | 7.8 | 12.2 | --- | --- | 42.5 | 36.7 | 35.2 | 48.7 | 37.4 |
| 2021 | 44.9 | 73.2 | 37.4 | 22.1 | 33.2 | 31.1 | 43.8 | 49.6 | 40.7 | 40.6 | 42.8 | 38.6 | 42.0 |
| Overall | **62.2** | **51.3** | **37.0** | **23.2** | **20.3** | **30.8** | **44.3** | **45.5** | **40.5** | **35.1** | **37.4** | **43.5** | **38.8** |

*Key: --- = Missing data either due to power outage or BAM 1022 Malfunctioning*
