# Supplementary material for: Air quality and attributable mortality among city dwellers in Kampala, Uganda: results from 4 years of continuous PM2.5 concentration monitoring using BAM 1022 reference instrument
Source: J Expo Sci Environ Epidemiol. 2024 Jun 15;35(2):288–93. doi: 10.1038/s41370-024-00684-9 (PMC11840866; doi:10.1038/s41370-024-00684-9)
Supplement: Supplementary file 3 — Supplementary Table 3 [file 41370_2024_684_MOESM3_ESM.docx]

Supplemental Table 3. Summary of annual means of PM_2.5_ concentration monitored by BAM-1022 for Kampala, Uganda 2018–2021

| Year | Annual Mean | Minimum PM2.5 | Maximum | Standard Deviation |
| --- | --- | --- | --- | --- |
| 2018 | 39.3 | 1.2 | 131.3 | 16.8 |
| 2019 | 34.9 | 12.3 | 93.3 | 15.0 |
| 2020 | 37.4 | 2.6 | 117.1 | 22.5 |
| 2021 | 42.0 | 12.2 | 162.9 | 18.9 |
| Overall | **38.8** | **1.2** | **162.9** | **18.6** |
